# Metabolic profile during pregnancy in BRISA birth cohorts of Ribeirão Preto and São Luís, Brazil

**DOI:** 10.1590/1414-431X202010253

**Published:** 2020-12-07

**Authors:** I.C. Rodrigues, C. Grandi, V.M.F. Simões, R.F.L. Batista, L.S. Rodrigues, V.C. Cardoso

**Affiliations:** 1 Departamento de Puericultura e Pediatria, Faculdade de Medicina de Ribeirão Preto, Universidade de São Paulo, Ribeirão Preto, SP, Brasil; 2 Argentine Society of Pediatrics, Buenos Aires, Argentina; 3 Departamento de Saúde Pública, Universidade Federal do Maranhão, São Luís, MA, Brasil

**Keywords:** Pregnancy, Cohort studies, Hypertension, Diabetes mellitus, Dyslipidemia

## Abstract

During pregnancy, metabolic changes that develop in women may increase the risk of diseases and conditions that may also harm the life of the growing fetus. The aim of the present study was to identify and compare the metabolic profile (MP) during pregnancy in two birth cohorts in 2010 in the cities of Ribeirão Preto (RP) and São Luís (SL), Brazil. Pregnant women (1393 in RP and 1413 in SL) were studied; information was obtained through questionnaires in addition to anthropometric, biochemical, and blood pressure measurements. Data are presented as means and proportions. To compare the characteristics of pregnant women in both cities, chi-squared and Student's *t*-tests were applied, with 5% significance level. Ribeirão Preto presented higher mean values than SL for pre-gestational body mass index (24.5 *vs* 23 kg/m^2^, P<0.001), systolic (108.4 *vs* 102.8 mmHg, P<0.001) and diastolic (65.9 *vs* 61.8 mmHg, P<0.001) blood pressure, total cholesterol (226.3 *vs* 213.7 mg/dL, P<0.001) and fractions, and glycemia (84.5 *vs* 80.2 mg/dL, P<0.001), except for triglycerides (P=0.135). Women from RP also showed higher rates of pre-gestational overweight and obesity compared with SL (40.1 *vs* 25.8%). In the present study, pregnant women in RP had a worse gestational metabolic profile than those in SL, with higher pre-gestational excess weight, indicating that nutritional transition was more advanced in the more developed city.

## Introduction

Brazil and several other Latin American countries have been experiencing a rapid demographic, epidemiological, and nutritional transition in the last twenty years (1). The marked increase in the prevalence of obesity in various population subgroups, including pregnant women, is noteworthy (1).

Pregnancy induces remarkable changes in maternal metabolism to support fetal demands (2,3). However, in some women the changes may be harmful and associated with adverse pregnancy outcomes; for example, gestational diabetes, hypertensive disorders, and preterm birth (3–5).

During pregnancy, high concentrations of total cholesterol, triglycerides, low-density lipoprotein cholesterol (LDL-C), and high-density lipoprotein cholesterol (HDL-C) are common (6). Serum lipid levels begin to increase from the 9th to the 13th week of gestation and peak at the 31st to 36th week (7). In addition, it's important to evaluate these levels during pregnancy, since lipid profile disorders are recognized to be involved in the pathophysiology of cardiovascular disease and diabetes (8–11).

Therefore, the identification of metabolic and inflammatory changes in pregnancy that may announce the risk of adverse outcomes is paramount. However, most epidemiological studies on the metabolic effects of pregnancy have a very small sample size and lack the possibility of replication (12). In addition, few studies addressed this issue in Brazilian pregnant women.

Thus, considering the scarcity in Brazilian literature and the relevance to maternal and child health, this study aimed to identify and compare the metabolic profile during pregnancy in two birth cohorts in the cities of Ribeirão Preto (RP) and São Luís (SL), Brazil.

## Material and Methods

### Study design

Secondary cross-sectional data obtained from two prenatal cohort studies, with a descriptive and analytical approach, was analyzed.

### Study population

This study was part of the “Etiological Factors of Preterm Birth and Consequences of Perinatal Factors in Child Health: Birth Cohorts in Two Brazilian Cities”, known as BRISA (Brazilian Ribeirão Preto and São Luis Birth Cohort) study. The main objective of BRISA was to evaluate new risk factors for preterm birth (neuroendocrine, immunoinflammatory, and medical intervention hypotheses), perinatal health indicators, and the impact on growth in two cohorts in the cities of RP and SL (13).

**Figure 1:**
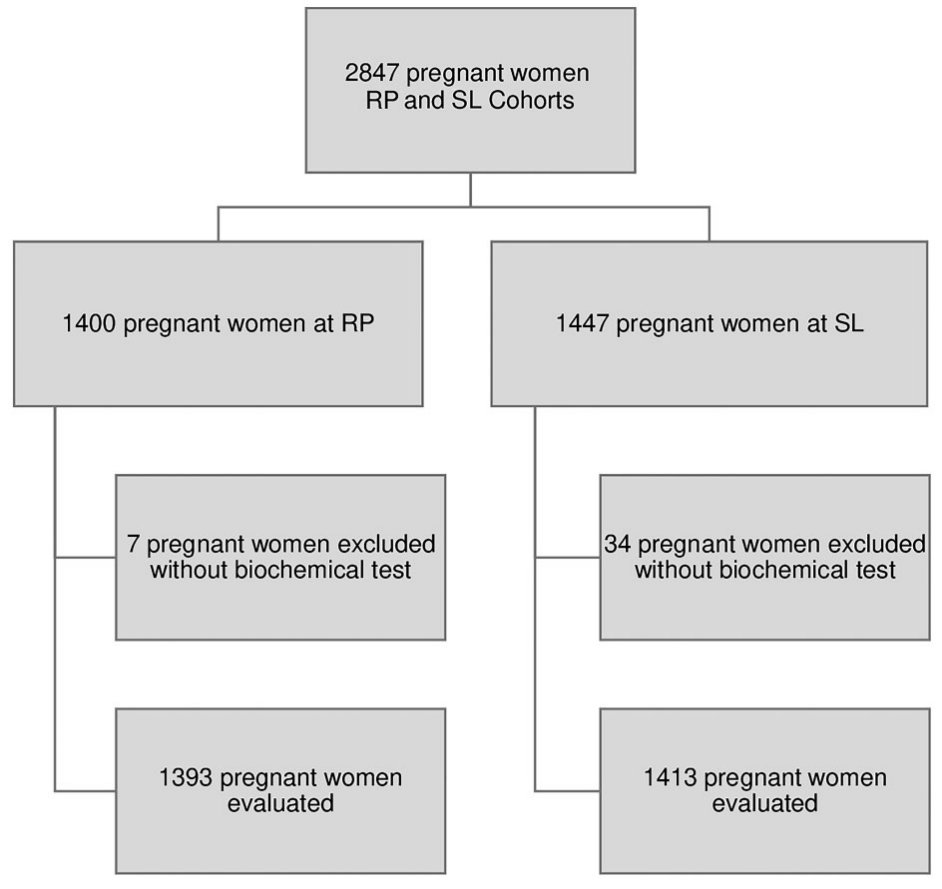
Flowchart of the study population. RP: Ribeirão Preto; SL: São Luis.

During 2010, pregnant women were recruited at public and private prenatal health facilities at less than the 20^+0^ week gestational age (GA). Inclusion criteria were women residing in RP and SL, with a single pregnancy and GA between 22^+0^-25^+6^ weeks (13). At this time, 1400 pregnant women in RP and 1447 in SL were evaluated. After excluding women without a biochemical test, the final sample consisted of 2806 pregnant women (1393 in RP and 1413 in SL) in the prenatal evaluation (Figure 1).

### Prenatal evaluation

Standardized questionnaires applied at 22^+0^ to 25^+6^ weeks GA included identification data, information on reproductive health, characteristics of the current pregnancy, age, education level, skin color, and marital status. Socioeconomic level categorized as A1/A2, B1, B2, C1/C2, and D/E, according to Brazilian Association of Research Companies (ABEP) (14) A1/A2 being the highest, prenatal visits, hypertension, diabetes, alcoholic and soft drink consumption, smoking, Block score (high-fat diet) (15), and type of delivery (vaginal, cesarean) were also evaluated. The short version of the International Physical Activity Questionnaire (IPAQ) was used to determine the physical activity level of the pregnant women, classified as vigorous or heavy, moderate, and light (16).

Non-fasting blood glucose and total cholesterol levels were measured using an enzymatic AA technique, HDL-C by single-phase AA plus colorimetric method, and triglycerides by GPO/PAP AA enzymatic method, all performed by the Wiener Lab CT600i autoanalyzer (Labinbraz Comercial LTDA, Brazil) at Ribeirão Preto Medical School, University of São Paulo laboratory. Blood pressure was measured two times using Omron 749 digital sphygmomanometer (OMRON Healthcare, Brazil) with a 15 min interval between measurements.

The maternal anthropometric measures were: self-reported pre-gestational weight (kg), and gestational weight (kg) and height (cm) measured in the prenatal evaluation (22^+0^-25^+6^ weeks) according to standardized techniques. Pre-gestational and gestational body mass index (BMI, kg/m^2^) were calculated according to the WHO classification (17).

The metabolic profile was classified according to the criteria proposed by Chatzi et al. (18) for metabolic syndrome including the following risk factors: BMI >30 kg/m^2^, fasting blood glycemia ≥100 mg/dL, systolic blood pressure ≥130 and/or diastolic blood pressure ≥85 mmHg, triglycerides ≥150 mg/dL, and HDL-C <50 mg/dL. Other biochemical markers evaluated were total cholesterol ≥250 mg/dL and LDL-C ≥130 mg/dL.

### Statistical analysis

Data are reported as means±SD or proportions, whichever was appropriate. Statistical tests included Student's *t*-test and chi-squared test. A P value <0.05 was considered significant. All statistical analyses were performed using the statistical package Stata, version 13.0 (Stata Corp. LP, USA).

### Ethical aspects

The databases were linked anonymously using encrypted individual health card numbers. The study was approved by the Research Ethics Committee of the Hospital das Clínicas and the Ribeirão Preto Medical School of the University of São Paulo (process number 11157/2008) and the Research Ethics Committee of the University Hospital of the Federal University of Maranhão (process number 4771/2008-30), and all participants freely signed the informed consent form.

Finally, we followed the STROBE (Statement for Reporting Observational Studies in Epidemiology) guidelines (19).

## Results

There were significant socioeconomic differences between the cities. Ribeirão Preto presented higher adolescent (14.0 *vs* 10.4%) and older pregnant women (10.1 *vs* 7.7%) rates, and lower level of schooling (27.5 *vs* 12.4%) than SL. On the other hand, non-white women were almost double in SL (83.8%) than RP (48.5%), but RP presented a higher percentage of women from the higher economic classes A1/A2 (1.5 *vs* 0.5%) (Table 1).

**Table 1:** Maternal sociodemographic characteristics and life styles during pregnancy. Comparison between the Ribeirão Preto and São Luís 2010 cohorts.

Characteristics	Ribeirão Preto (n, %)	São Luís (n, %)	P
Sociodemographic			
Age (years)			<0.001
20-34	1040 (75.9%)	1132 (81.9%)	
<20	192 (14.0%)	144 (10.4%)	
≥35	138 (10.1%)	106 (7.7%)	
Skin color			<0.001
White	700 (51.5%)	224 (16.2%)	
Non-white	658 (48.5%)	1157 (83.8%)	
Maternal education (years)			<0.001
≥12	114 (8.4%)	172 (11.9%)	
9-11	874 (64.1%)	1091 (75.7%)	
≤8	375 (27.5%)	179 (12.4%)	
Marital status (partner)			0.360
Yes	1138 (81.5%)	1160 (80.2%)	
No	258 (18.5%)	287 (19.8%)	
Socioeconomic level (ABEP)			<0.001
A1/A2	20 (1.5%)	7 (0.5%)	
B1	55 (4.2%)	38 (2.7%)	
B2	293 (22.3%)	176 (12.7%)	
C1/C2	794 (60.3%)	933 (67.6%)	
D/E	154 (11.7%)	226 (16.3%)	
Life style during pregnancy			
Alcoholic drink consumption			<0.001
Yes	344 (25.1%)	181 (13.1%)	
No	1026 (74.9%)	1021 (86.9%)	
Soft drinks consumption			<0.001
Yes	1107 (79.3%)	837 (57.9%)	
No	288 (20.7)	609 (42.1%)	
Smoking			<0.001
Yes	174 (12.7%)	38 (2.7%)	
No	1196 (87.3%)	1344 (97.3%)	
Block score altered (>27)			0.600
Yes	396 (28.4%)	398 (27.5%)	
No	998 (71.6%)	1048 (72.5%)	
Physical activity			<0.001
Sedentary	244 (17.5%)	95 (6.6%)	
Light	455 (32.7%)	487 (33.6%)	
Moderate	404 (29.1%)	601 (41.5%)	
High	288 (20.7%)	264 (18.3%)	

Chi-squared test was used. ABEP: Association of Research Companies.

Compared with SL, Ribeirão Preto pregnant women had a higher intake of alcoholic drinks (25.1 *vs* 13.1%) and soft drinks (79.3 *vs* 57.9%) during pregnancy, were more sedentary (17.5 *vs* 6.6%), and smoking rates were almost five times greater (12.7 *vs* 2.7%). Altered Block scores showed no significant differences (Table 1).

RP showed significantly higher rates of pre-gestational overweight/obesity (40.1 *vs* 25.8%), more prenatal visits (94.8 vs 82.9%), and gestational diabetes (5.3 *vs* 2.7%), but lower rates of gestational hypertension (14 *vs* 16.9%) and cesarean section (40.4 *vs* 50.2%) than SL (Table 2).

**Table 2:** Reproductive and obstetric characteristics. Comparison between the Ribeirão Preto and São Luís 2010 birth cohorts.

Characteristics	Ribeirão Preto (n, %)	São Luís (n, %)	P
Pre-gestational BMI (kg/m^2^)			<0.001
18.5-24.9	733 (52.7%)	929 (66.3%)	
<18.5	100 (7.2%)	110 (7.9%)	
25-29.9	364 (26.2%)	277 (19.7%)	
≥30	193 (13.9%)	86 (6.1%)	
Prenatal control (≥ 6 visits)			<0.001
Yes	1245 (94.8%)	1077 (82.9%)	
No	69 (5.2%)	221 (17.1%)	
Gestational diabetes			
Yes	73 (5.3%)	37 (2.7%)	
No	1297 (94.7%)	1344 (97.3%)	
Gestational hypertension			0.038
Yes	192 (14.0%)	233 (16.9%)	
No	1178 (86.0%)	1148 (83.1%)	
Type of delivery			<0.001
Vaginal	817 (59.6%)	689 (49.8%)	

Chi-squared test was used. BMI: body mass index.

All the anthropometric and blood pressure values were significantly higher in RP compared with SL pregnant women (P<0.001) (Table 3).

**Table 3:** Anthropometric and blood pressure profiles of pregnant women in Ribeirão Preto and São Luís 2010 birth cohorts.

Anthropometric and blood pressure profile	Ribeirão Preto			São Luís			P
	n	Mean	SD	n	Mean	SD	
Pre-gestational weight (kg)	1390	63.6	14.5	1418	56.3	10.9	<0.001
Height (m)	1400	1.60	0.06	1429	1.56	0.05	<0.001
Pre-gestational BMI (kg/m^2^)	1390	24.5	5.2	1402	23.0	4.1	<0.001
Gestational BMI	1396	27.1	5.2	1419	25.5	4.1	<0.001
Systolic blood pressure (mmHg)	1398	108.4	9.8	1413	102.8	10.1	<0.001
Diastolic blood pressure (mmHg)	1399	65.9	7.6	1413	61.8	8.1	<0.001

Student's *t*-test was used. BMI: body mass index.

The means (SD) of the biochemical tests were 220 (40.5) mg/dL for total cholesterol, 118.9 (36.1) mg/dL for LDL-C, 65.5 (13.6) mg/dL for HDL-C, 176.7 (66.9) mg/dL for triglycerides, and 82.4 (18.3) mg/dL for glycemia. When the two populations were compared, all biochemical values were statistically higher in RP, except for triglycerides (Table 4).

**Table 4:** Biochemical profile of pregnant women in Ribeirão Preto and São Luís 2010 birth cohorts.

Biochemical profile (mg/dL)	Ribeirão Preto			São Luís			P
	n	Mean	SD	n	Mean	SD	
Total Cholesterol	1393	226.3	41.4	1412	213.7	38.6	<0.001
LDL Cholesterol	1393	121.3	34.7	1413	116.5	37.3	<0.001
HDL Cholesterol	1393	68.1	14.1	1407	62.8	12.5	<0.001
Triglycerides	1393	178.6	68	1413	174.8	65.8	0.135
Glycemia	1393	84.5	19.1	1413	80.2	17.2	<0.001

Student's *t*-test was used. SD: standard deviation; LDL: low density lipoprotein; HDL: high density lipoprotein.

When applying the cutoff points for metabolic syndrome (MS) indicators according to Chatzi criteria (18), we observed that altered blood pressure (4%), altered glycemia (16.5%), and obesity (13.6%) were higher in RP than SL (2, 9.9, and 5.6%, respectively). Only altered rates of HDL-C were higher in SL pregnant women (13.9%), whereas no differences were observed between the cities regarding altered triglycerides (Table 5).

**Table 5:** Altered metabolic syndrome components in Ribeirão Preto and São Luís 2010 birth cohorts.

Altered metabolic syndrome components	Ribeirão Preto (n, %)	São Luís (n, %)	P
Triglycerides (≥150 mg/dL)	855 (61.4%)	841 (59.5%)	0.314
Blood pressure (≥130/≥85 mm Hg)	56 (4.0%)	29 (2.0%)	0.002
HDL cholesterol (<50 mg/dL)	104 (7.5%)	196 (13.9%)	<0.001
Glycemia (≥100 mg/dL)	230 (16.5%)	141 (9.9%)	<0.001
Obesity (BMI >30 kg/m^2^)	189 (13.6%)	79 (5.6%)	<0.001

Chi-squared test was used. HDL: high density lipoprotein; BMI: body mass index.

## Discussion

Compared with SL, pre-gestational BMI, blood pressure, and all biochemical tests were significantly higher in RP, except for triglycerides. Similar findings were observed in a previous study for cholesterol, triglycerides, HDL-C, and LDL-C in eutrophic pregnant women between 24- to 29-weeks GA (20). The normal physiology of pregnancy includes insulin resistance, increasing adipose tissue, hyperlipidemia, an increase in blood pressure, and activation of the inflammatory cascade. During pregnancy, approximately 6-8% of women develop gestational diabetes and 3-5%, hypertension (21). Pregnancy contributes significantly to weight gain in women; data reveal that 42% of women gain weight above the Institute of Medicine's (IOM) recommendations during pregnancy, and are more likely to develop MS than those without a history of excessive weight gain (22).

The definitions of metabolic factors (MF) associated with non-transmissible diseases in pregnancy are also controversial because the criteria for detecting those factors overlap with the physiological changes of pregnancy. Women with pre-gestational altered MF are more likely to develop complications during pregnancy, such as pre-eclampsia, eclampsia, gestational diabetes, and coma (23).

The unfavorable metabolic profile of pregnant women in RP can be attributed to the higher consumption of beverages during pregnancy, a four-fold incidence of smoking, more smokers who smoked more than 10 cigarettes per day (heavy smoker), and lower levels of physical activity. On the other hand, the absence of difference in the Block score, which evaluates a high-fat diet, can be attributed to limited sensitivity of the method. It can also be speculated that greater access to food can be associated with a higher socioeconomic level in RP compared to SL.

The development of an unfavorable metabolic profile is highly related to changes in the population's nutritional profile, due to nutritional transition. An unhealthy nutritional transition (increased malnutrition - energy-dense foods) can lead to shortened growth (stunting) and weight gain in children, adolescents, and adults, resulting in higher BMI and worse health outcomes throughout the life cycle (24).

The present study demonstrated that pregnant women in RP presented disturbing levels of overweight and obesity, already in the pre-conception period, in contrast to women in SL, a fact that allows us to speculate whether the nutritional transition seems to be more advanced in the more developed city.

Another explanation for the observed paradoxes of the more developed city (RP) having higher rates of low schooling and lower C-section rates are the convenience sampling.

In an Argentine study, total cholesterol of 159.8 (32.5) mg/dL, LDL-C of 78.0 (26.3) mg/dL, HDL-C of 57.6 (11.5) mg/dL, and triglycerides of 89.9 (33.8) mg/dL were observed in pregnant women in the first trimester (n=248) (25). In our study, all values were much higher, reflecting the nutritional status of pregnant women at the beginning of pregnancy and their lifestyle; also, all biochemical values were significantly higher in RP, except for triglycerides (Table 3).

The greater rates of gestational diabetes and altered non-fasting glycemia in RP agrees with a previous study (26) on the need to investigate pre-gestational metabolic syndrome. Maternal health in association with nutritional status is the most important factor related to pregnancy outcomes and perinatal morbidity (27).

A limitation of the study was the measure of glycemia without fasting, because it was a study involving pregnant women, in an evaluation that took an average of 4 h, which may have over- or underestimated the results. Nonetheless, the main study objective was to compare the two cities regarding the metabolic profile.

The main study strength was the comparison of data from two cohorts with marked socioeconomic and nutritional transition contrasts, which allowed better detection of differences in metabolic markers that pose risks to the mother and her fetus. These risks not only have a long-term impact on maternal health but are also responsible for many abnormalities in children like the risk of metabolic syndrome and cardiovascular disease induction (28).

In conclusion, RP had a worse gestational metabolic profile than SL in pregnant women, with higher pre-gestational excess weight, indicating a nutritional transition in the more developed city. The components of the metabolic factors associated with non-transmissible diseases must be diagnosed early in order to offer the opportunity for health professionals to intervene, with the aim of promoting health and preventing complications, for both the mother and her newborn.
